# The Contamination of Microplastic Debris in Blue Swimming Crab *Portunus pelagicus* (Linnaeus, 1758) from Artisanal Fisheries in the Eastern Gulf of Thailand

**DOI:** 10.3390/toxics13100813

**Published:** 2025-09-24

**Authors:** Poratape Jendanklang, Chakhrit Ruengsorn, Shettapong Meksumpun, Pattira Kasamesiri

**Affiliations:** 1 Department of Fisheries, Rayong Marine Fisheries Research and Development Center, Rayong 21160, Thailand; poratape.j@ku.th; 2 Department of Marine Science, Faculty of Fisheries, Kasetsart University, Bangkok 10900, Thailand; ffisspm@ku.ac.th; 3 Division of Fisheries, Faculty of Technology, Mahasarakham University, Maha Sarakham 44150, Thailand; pattira@msu.ac.th

**Keywords:** crab gillnet, fishing gear, microplastic pollution, microplastic migration

## Abstract

Microplastics have become a significant concern for human health, primarily because aquatic animals can ingest these particles, which then enter the human food chain. Crabs (*Portunus pelagicus*) were collected along the coastline of Rayong Province in January, April, and August 2024. Crabs were then examined for MP contamination. Our results revealed that MPs were present at all sampling sites, with a detection rate of 62.5% in external body parts and 72.2% in internal body parts. The gut was the most contaminated tissue, followed by the gills, while no MPs were found in the hepatopancreas or muscle tissues. Although overall MP detection and contamination levels were similar across sites, significant differences in abundance were observed between seasons (*p* < 0.05), with August showing the highest contamination levels. Polyethylene terephthalate glycol was the most common polymer detected, followed by nylon, polypropylene, polyethylene, polystyrene, and polyester. Anthropogenic and fishing activities contribute significantly to MP pollution in these crabs. Fibers from household laundry, followed by damaged fishing gear, are major sources of MP pollution. Enhancing the quality and durability of fishing equipment is crucial to reducing the amount of abandoned fishing gear that may be ingested by marine organisms, while the proper collection and management of discarded gear in the ocean should also be emphasized.

## 1. Introduction

Plastic debris is one of the most serious pollutants contributing to ocean pollution. It is one of the greatest environmental challenges of the 21st century. Recently, Thailand was rated as having the sixth highest rate of plastic flow into oceans in the world [1]. Thailand is also recognized among the nations with challenges in plastic waste management [2]. The presence of plastic debris of varying sizes, ranging from macroplastics to microplastics, poses severe threats to marine ecosystems and biodiversity [3]. Plastic debris such as fishing gear can lead to entanglement and ingestion by organisms [4]. It can cause injury, abnormal mobility, and mortality [5]. Rapid economic and population expansion, lifestyle changes, ineffective governance practices, and insufficient awareness and knowledge about proper waste disposal can contribute to plastic pollution in our environment [6]. The origins of plastic debris are usually terrestrial and marine. The mismanagement of waste and urban runoff are terrestrial sources of approximately 80% of plastic debris [7,8], while ocean-based sources mostly include damaged and discarded fishing gear, litter from vessels near shore, and offshore industrial activities [9]. Debris can be transported by wind and currents in the ocean, which can lead to it being spread across the world, including remote areas [10].

Microplastics (MPs) are plastic particles less than 5 mm in size that originate from the fragmentation of larger plastics produced for products like cosmetics and synthetic textiles [11,12,13]. Owing to their small size, these particles can contaminate various ecologies, such as water and sediment in watercourses [14], water and beach sand along shores [15], mangrove sediment [16], and sediment in estuarine systems [17], and they can mix with indoor dust [18] and airborne particulate matter (PM_2.5_) [19]. Moreover, they can accumulate in a wide range of marine organisms, including zooplankton [20], fish larvae [21], bivalves and gastropods [22], pelagic and demersal fishes [23], sea turtles [24], sea birds [25], and cetaceans [26]. When accumulated in organisms, MPs can cause physical harm and blockages, interfere with feeding and respiration, cause malnutrition, and result in exposure to toxic chemicals leached from them [27,28]. They can associate with pollutants and be transferred up through the food chain, potentially leading to adverse health effects [29]. These harmful MP particles not only affect the health of aquatic animals [30], but they can also pose a risk to human health through the consumption of MP-contaminated marine life [31,32].

Blue swimming crab (*Portunus pelagicus*) is a carnivorous bottom-dweller, mainly preying on mollusks, invertebrates, and bony fish [33]. This crab species is found spanning wide-ranging habitats with high commercial importance in various regions, including the Indo-West Pacific region with coastal waters from India, Australia, Africa, and Asia, with particular in Thailand [34]. This crustacean is a commercially important marine species found in various coastal regions in both the Andaman Sea and the Gulf of Thailand, including Rayong Province, which is located in the Eastern Gulf of Thailand. Marine capture by artisanal fisheries in Thailand in 2023 showed the volume of aquatic animals in crab category was 41,442.02 tons, with blue swimming crabs at 34,899.75 tons, accounting for 84.21% of the total catch. In addition, 77.98% of blue swimming crabs were caught by crab gillnets from artisanal fisheries [35]. Crab gillnet is the common fishing gear for catching this economic crab in this region by local fishermen [36]. According to the literature on MPs in crabs in Thailand, recent studies have detected MPs in blue swimming crabs from coastal Chonburi province [37] and Thai vinegar crabs from a mangrove area in Samut Prakan province [38], revealing pervasive plastic pollution. Marine ecosystems in the Rayong province were threatened by MP pollution, as evidenced by the presence of MPs in the environment and organisms around the Rayong province [15,23,39]. This contamination underscores the importance of implementing strategies to mitigate plastic pollution and protect marine and human health in this region.

Due to the characteristics and extent of MPs, the contamination in blue swimming crabs caught from artisanal fisheries remains unclear. Our study aims to study (i) the detection rate of MPs, (ii) the abundance and composition of MPs in tissue, and size and sex of crabs, and (iii) the characteristics of MP contamination in blue swimming crabs along four sampling sites seasonally in the Rayong province, an important fishing ground of blue swimming crabs in the Eastern gulf of Thailand. To reach this purpose, we collected blue swimming crab samples and dissected four parts of the tissue for extracting MPs from internal and external bodies, including interviewing local fishermen by gathering fishing ground data with a semi-structured interview method to estimate the accumulation of MPs between season and sampling sites to clarify the situation of MP debris in organisms in this area.

## 2. Materials and Methods

### 2.1. Study Area and Sampling Sites

**Figure 1:**
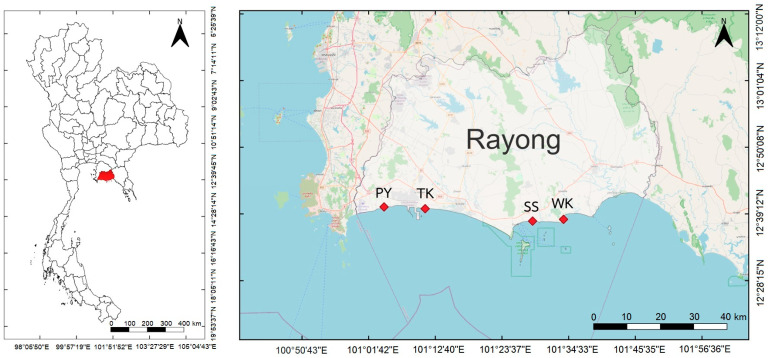
Location of sampling sites along the Rayong province.

Rayong province is located on the eastern coast along the Gulf of Thailand, which is characterized by its industrial, agricultural, and tourism activities. The coastal area of this province spans approximately 100 km [40] and harbors diverse fisheries, including the fishing of blue swimming crab (*P. pelagicus*) by artisanal fisheries, especially crab gillnet. Sample collections were conducted in January, April, and August 2024, which represented the northeast monsoon, transition monsoon, and southwest monsoon, respectively. Crab samples were collected from four sampling sites, including Phayun (PY), Takuan (TK), Suanson (SS), and Wangkaew (WK), along the coast of Rayong province (Figure 1).

### 2.2. Fisheries Data Gathering

Fisheries data were collected by interviewing local fishermen with a semi-structured interview method using an interview questionnaire form, which is provided in Figure S1. Details of the interview questionnaire form included vessel and fishermen data, characteristics of fishing gear with lifespan and materials, method of fishing operation, fishing ground area, environmental data, species composition, and total catch. This study focused on local fishermen who collected crab samples and were followed up throughout the study to ensure crab samples were caught in their fishing community area. The sample size of interviewed and followed-up fishermen throughout the study was twelve samples (N = 12). However, data on characteristics of fishing gear, materials, lifespan, and fishing grounds of blue swimming crabs by crab gillnet were only used for further discussion.

### 2.3. Sampling Method

Blue swimming crabs (*P. pelagicus*) were collected from small-scale fisheries in Rayong province from 4 stations, with 12 individuals per station. A total of 48 individuals will be sampled per season, and a total of 144 individuals of *P. pelagicus* will be used throughout the study. The sampling stations include Phayun, Takuan, Suanson, and Wangkaew. Crab samples were categorized into two size groups: small (6 crabs: 3 males, 3 females) and large (6 crabs: 3 males, 3 females). Sizes of crabs were categorized by sold size. Before analyzed, all samples were measured for outer carapace width and carapace length, and weighed. Crab samples were stored at −20 °C to preserve the samples before dissection and extracting MPs in the laboratory.

### 2.4. Microplastics Digestion and Separation

**Figure 2:**
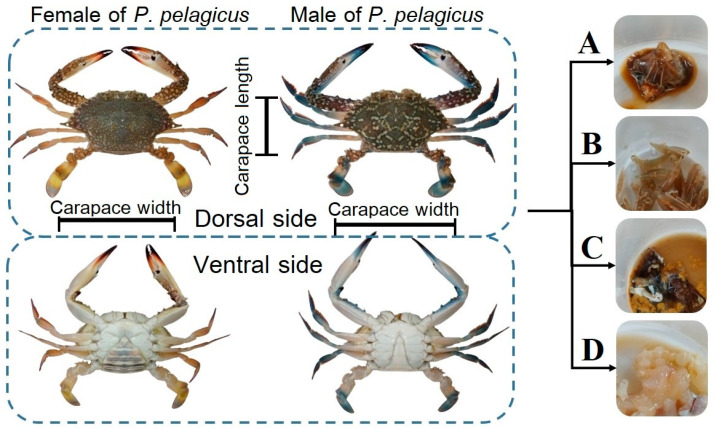
Dissecting by used part: (A) gut, (B) gill, (C) hepatopancreas, and (D) muscle.

The MP separation protocol was adapted from the method developed by the National Oceanic and Atmospheric Administration (NOAA) [41]. Crab samples were individually washed with 100 mL of distilled deionized water for external MP sampling. Washed water from the external body of crab samples individually was defined as external MP contamination, which was digested, separated, and filtered using the same procedure as internal tissues. Procedural and airborne blanks controlled by distilled deionized water were tested in parallel for each group of crabs categorized by sampling site and sampling time using the same procedure as internal tissues. All crabs were dissected to analyze internal contamination, focusing on gut, gill, hepatopancreas, and muscle tissue (excluding muscle tissue of walking and swimming legs) (Figure 2). Each tissue part was weighed separately and subjected to the wet peroxide oxidation (WPO) method. This involved adding 30% hydrogen peroxide (Qrec, Auckland, New Zealand) and 1% potassium hydroxide (Qrec, Auckland, New Zealand) in a 9:1 ratio, increasing the temperature to 75 °C while stirring until organic gas bubbles disappeared, and then allowing 24 h for the removal of organic matter. After completing the WPO method, MPs were separated using a density separation procedure. NaCl solution was added, stirred with a glass rod, and left to settle for 24 h to collect the supernatant, and the settled solution was also collected to prepare filtration, which will recover higher-density polymers. The supernatant and settled solution were filtered separately through a 47 mm diameter glass microfiber filter (Whatman grade GF/C pore size: 1.2 µm) using a vacuum system. The filter paper was dried in an oven at 60 °C and stored in a Petri dish glass with a glass lid for later visualization and identification.

### 2.5. Microplastics Visualization and Identification

MPs on filter paper were analyzed for quantity, size, shape, and color using a stereo microscope with total magnification used as 15× (Olympus SZ51, Hamburg, Germany). A hot needle test was used to distinguish between plastic and non-plastic materials [42]. Although the hot needle test is a common method for screening plastic and non-plastic materials, it has several limitations that reduce its discriminative power. These limitations can lead to false positives and negatives and could affect results, making them less reliable than advanced methods like FTIR. However, this technique was only used as a preliminary step before identification by FTIR. MP sizes were measured with ImageJ version 1.50i and categorized into three ranges: 15–150 µm, 150–330 µm, and 330–5000 µm. Particle shapes were classified as fiber, fragment, film, and net. The study identified MPs in four colors (black, blue, red, and green); clear items were reported. MP particles were analyzed, 10% from total particles founded in this study. Polymer classifications were confirmed using Fourier-transform infrared spectrophotometry (FTIR Type II; Perkin Elmer, High Wycombe, UK).

### 2.6. Data Analysis

Average MP abundance was calculated in terms of items/individual and items/g wet weight with standard deviation values. The Shapiro–Wilk test was used to check for normal distribution (*p* < 0.05). Due to the non-normality of the data, nonparametric statistical methods were applied. The Kruskal–Wallis H test and pairwise comparisons were used to analyze the variations in MP abundance across different sampling sites and seasons. The Microplastics Detection Rate (MDR) was also calculated by the quantity of crab samples, which were contaminated in terms of individuals from all samples and categorized by sampling sites, seasonality, size groups of crab, type sex of crab, and part of tissues by Equation (1):

MDR (%) = C_c_/N_c_ × 100, (1)

where:

MDR = Microplastics Detection Rate (%);

C_c_ = total contaminated crabs (individuals);

N_c_ = total crab samples.

### 2.7. Quality Control

Procedural blank controls were used by distilling deionized water. These processes were tested as three blank parallel controls for each group of crabs categorized by sampling site and sampling time using the same procedure as internal tissues. Procedural blank controls were thirty-six (N = 36). Airborne blank controls were also tested by leaving three Petri dishes with open cover lids in the laboratory throughout the process, then rinsing with 100 mL of distilled deionized water in the Petri dish to test airborne contamination with the same method as the procedural blank controls (N = 36). Results from all procedural and airborne blank controls showed no MP contamination in our laboratory. The blank-corrected counts were equal to raw MPs counts, as no MP contamination was detected in all blanks.

## 3. Results and Discussion

### 3.1. Blue Swimming Crab Fishing Ground

**Figure 3:**
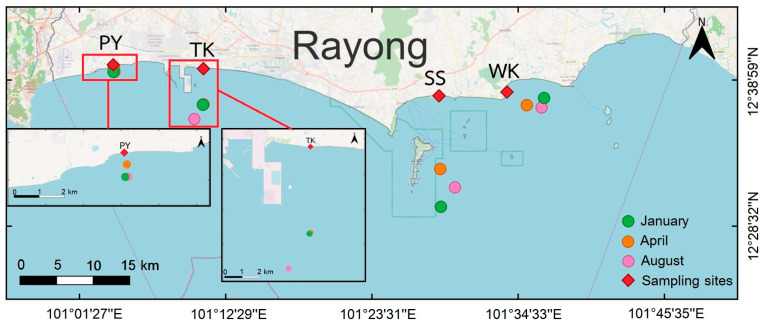
Crab gillnet fishing grounds at four sampling sites in Rayong province. Green, orange, and pink circles indicate January, April, and August, respectively; red diamonds mark the sampling sites.

Our data showed crab gillnet is the most common fishing gear for blue swimming crab fisheries, especially artisanal fisheries in this study area. Crab gillnets in Rayong province are specifically designed with larger mesh sizes around 3 inches to catch blue swimming crabs, which are made from durable materials like nylon and polyethylene to withstand the coastal environment. Distances from coast to fishing grounds and water depths were collected by a semi-structured interview method from local fishermen for four locations: PY, TK, SS, and WK. In PY, distances were short (0.5–1 km) with water depths of 2–4 m. TK had distances around 5–7 km with deeper waters (15–18 m). SS showed a greater variability with distances of 10–15 km and water depths of 8–19 m. WK had distances ranging between 3–5 km and water depths of 8–14 m (Figure 3).

### 3.2. Crab Characteristics

A total of 144 individuals of *P. pelagicus* were caught by artisanal fisheries from four sampling sites in Rayong province. The characteristics of the blue swimming crabs in this study were shown in Table 1.

### 3.3. Microplastics Detection Rate

**Figure 4:**
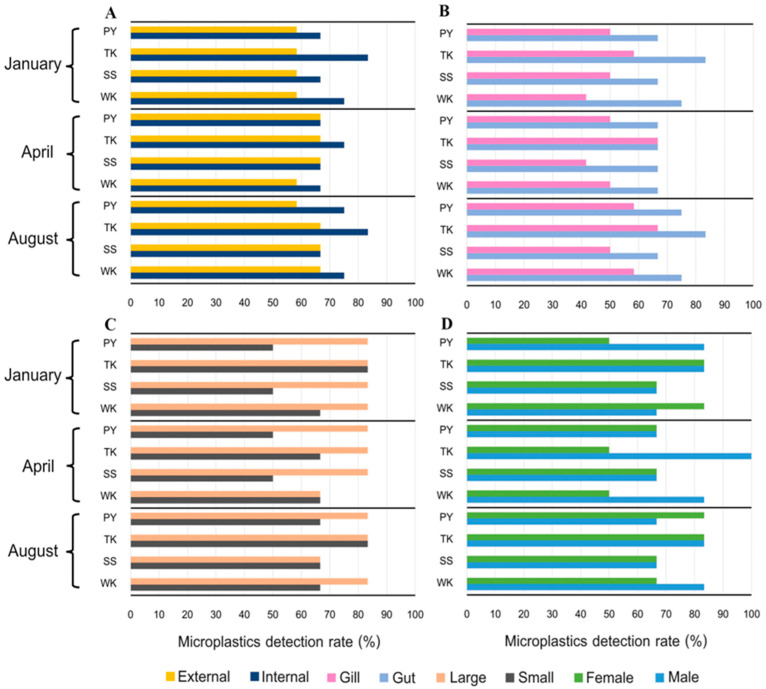
MP detection rate in blue swimming crab (%) in Rayong province categorized by (**A**) internal and external detection rate (%), (**B**) tissue (%), (**C**) size (%), and (**D**) sex in Rayong province: Phayun—PY, Takuan—TK, Suanson—SS, Wangkaew—WK.

MPs were detected in *P. pelagicus* across all sampling sites and seasons in Rayong province, with an overall detection rate of 72.2% (104 individuals). TK station had the highest detection rates at 83.3%, 75.0%, and 83.3% in January, April, and August 2024, respectively (Figure 4). When compared to other studies, the overall detection rate of MPs in crabs from China (89.3%) showed a higher detection rate than our study [43]. However, a consistent detection rate across seasons and sampling sites indicates contamination is a persistent issue, not limited to specific times and areas in Rayong province. MPs were found in all size groups, with larger crabs having a higher detection rate (80.6%) compared to smaller crabs (63.9%), showing a significant difference (Kruskal–Wallis, *p* < 0.05). When considering specific tissues, guts had a higher detection rate (71.5%) than gills (53.5%), with a significant difference between them (Kruskal–Wallis, *p* < 0.05). No MPs were detected in the hepatopancreas and muscles of crabs, at least not in detectable amounts. While there was no significant difference in MP detection rates between male and female crabs, males showed a slightly higher detection rate (76.4%) compared to females (68.1%).

### 3.4. Abundance of MPs

**Figure 5:**
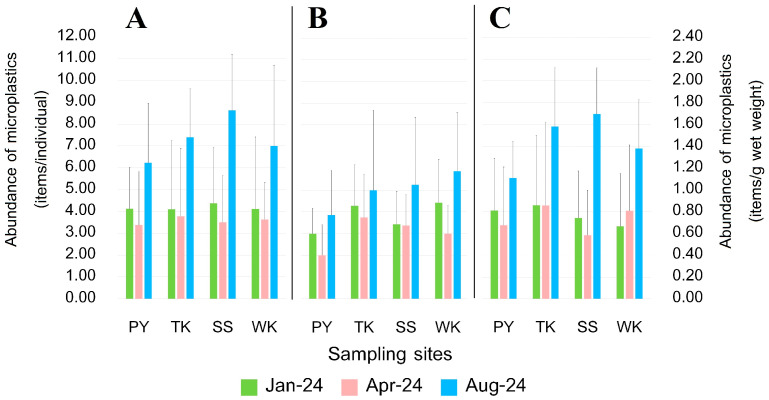
Average abundance of MP contamination of *P. pelagicus:* (**A**) internal contamination in terms of items/individual, (**B**) external contamination in terms of items/individual, and (**C**) internal contamination in terms of items/g wet weight along Rayong province: Phayun—PY, Takuan—TK, Suanson—SS, Wangkaew—WK.

MP contamination in blue swimming crabs varied between internal and external samples, with 533 items found internally and 356 items found externally. The average abundance of MPs within the crabs ranged from 3.38 ± 2.45 to 8.63 ± 2.56 items/individual across all sampling sites and seasonal, and from 0.58 ± 0.41 to 1.70 ± 0.42 items/g wet weight for January, April, and August 2024 (Figure 5). For external contamination, the average abundance ranged from 2.00 ± 1.41 to 5.88 ± 2.70 items/individual over the same periods. The highest internal contamination was recorded at SS in August 2024, with an average of 8.63 ± 2.56 items/individual, while the lowest was found at PY in April 2024, with 3.38 ± 2.45 items/individual. Externally, the highest contamination was observed at WK in August 2024, with 5.88 ± 2.70 items/individual, and the lowest at PY in April 2024, with 2.00 ± 1.41 items/individual. PY is characterized by few beachside seafood restaurants, hotels, and small-scale fishing communities. On the other hand, TK is located near the Nam Hu canal within the industrial zone of Map Ta Phut Industrial Estate, with a densely populated area, and features beachside seafood restaurants, hotels, and small-scale fisheries communities. The SS site is located in a densely populated coastal community, where the mouth of the canal discharges land-based waste into the sea. In addition, this area is a major tourist destination, with intensive recreational activities, and it also hosts a pier that serves as the main transit point for tourists traveling to Samed Island. These factors likely contribute to the high abundance of microplastic debris observed at this station. Although WK has some tourist activities, including hotels and beachside seafood restaurants similar to other sites, the residential area around WK is less densely populated than SS. These varying environmental conditions, levels of anthropogenic activities, tourist activities from shore [44], and fishing activities around fishing grounds could influence the extent of MP contamination in blue swimming crabs. However, a spatial distribution showed no significant differences in MP contamination between sampling sites. In contrast, the seasonal variation was found to be significant, with the highest contamination in August for both internal and external samples (Kruskal–Wallis, *p* < 0.05). The season also plays a critical role in MP abundance. These differences could be attributed to several factors, including the variations in fishing activity, water currents, and weather patterns in each sampling time that affect the abundance and breakdown of plastic debris and fishing gear. During August 2024, which represents the wet season or southwest monsoon, with a high rainfall and runoff, more MP debris from terrestrial areas might be washed into nearby rivers, increasing MPs concentrations, then flow out into the ocean [14]. When compared to other studies, our study’s results were higher than *P. pelagicus* from Chonburi province, Thailand (0.72 items/individual) [37], *Portunus trituberculatus* from Liaohe Estuary, China (1.33 items/individual) [45], *Panopeus herbsti* from the Indian River Lagoon system, USA (4.2 items/individual) [46], *Carcinus aestuarii* from the Northern Adriatic coast, Italy (1.1 ± 0.7 items/individual) [47], and *Emerita analoga* from the Beaches of California, USA (1.39 ± 0.79 items/individual) [48], and, in terms of items/g wet weight, *Portunus sanguinolentus* from Gujarat state, India (0.67 ± 0.62 items/g wet weight) [49].

#### 3.4.1. Crab Tissue

**Figure 6:**
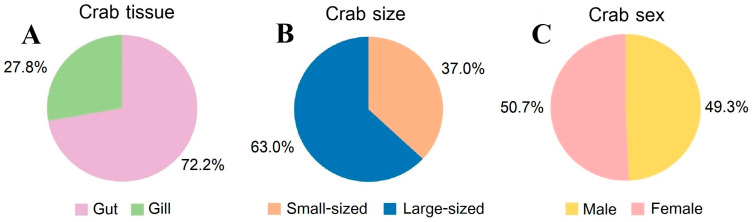
Internal composition of MP contamination in *P. pelagicus* categorized by (**A**) tissue (%), (**B**) size (%), and (**C**) sex (%) in Rayong province.

MP contamination can expose organisms in four main ways: direct contact, ingestion, inhalation, and entanglement [50]. MPs were found in the gut and gill of crabs, with no contamination detected in the hepatopancreas and muscle tissues (Figure 6A). The gut was the primary site of MP accumulation, accounting for 72.2% of the total contamination, while gill tissue contributed 27.8%. The difference in MP contamination between the gut and gill tissues was found to be significant (Kruskal–Wallis, *p* < 0.05), which indicates the gut plays a major role in MP ingestion and retention. Similarly to previous studies, MPs in *P. sanguinolentus* [49] and *Carcinus maenas* [51] were recorded to be higher in guts than in gills. The role of the gut in processing food makes it a likely location for the accumulation of ingested MPs. Moreover, crabs use their claws to break fishing nets to free themselves. Many of these severed lines, which are fiber and net, can be ingested by crabs and enter their digestive system [43]. In contrast, the gill showed less accumulation. The primary function of the gill is respiration by water flowed through their body, which might make it less prone to high levels of MP accumulation compared to the gut. The absence of MPs in the hepatopancreas and muscle tissues indicates that these organs are less involved in the direct processing and accumulation, and translocate effectively from the gut to these tissues.

#### 3.4.2. Crab Size

An analysis of crabs by size revealed a significant disparity in MP contamination between small and large crabs. Small crabs accounted for 37.0% of the contamination, whereas large crabs made up 63.0% (Figure 6B). This difference was significant across all sampling sites and seasons (Kruskal–Wallis, *p* < 0.05), indicating that larger crabs are more likely to have higher levels of MP contamination. According to a previous study, the size of *P. pelagicus* was found to have a small correlation to the abundance of MPs in their digestive tract [52]. However, our result shows a high Spearman’s rank correlation between crab size and the abundance of MPs that are contaminated in the internal body (rs = 0.434, *p* < 0.01) and external body (rs = 0.724, *p* < 0.01). This might be due to larger crabs having a greater food intake and feeding activity when compared to smaller crabs. Crabs require food to retain their metabolic demands in their body [53], which, consequently, increases their exposure to MPs in their habitat and environment.

#### 3.4.3. Crab Sex

When comparing male and female crabs, MPs were contaminated in females at a slightly higher rate than males, 49.3% in males and 50.7% in females (Figure 6C), with no significant difference in MP contamination between sexes across any sampling sites or seasons, which indicates that sex does not play a significant role in the differential accumulation of MPs. However, in a previous study in *P. pelagicus*, female crabs were also found to be slightly more contaminated than males [37], in contrast to MPs in *P. sanguinolentus*, which were found to be higher in males than in females [49]. The sex of crab samples and MP contamination with long-term effects in their internal tissue might be studied further in the future. As a result, the size and tissue parts are more critical in determining the levels of contamination rather than sex-specific differences.

### 3.5. Characteristics of MPs

#### 3.5.1. MP Size

**Figure 7:**
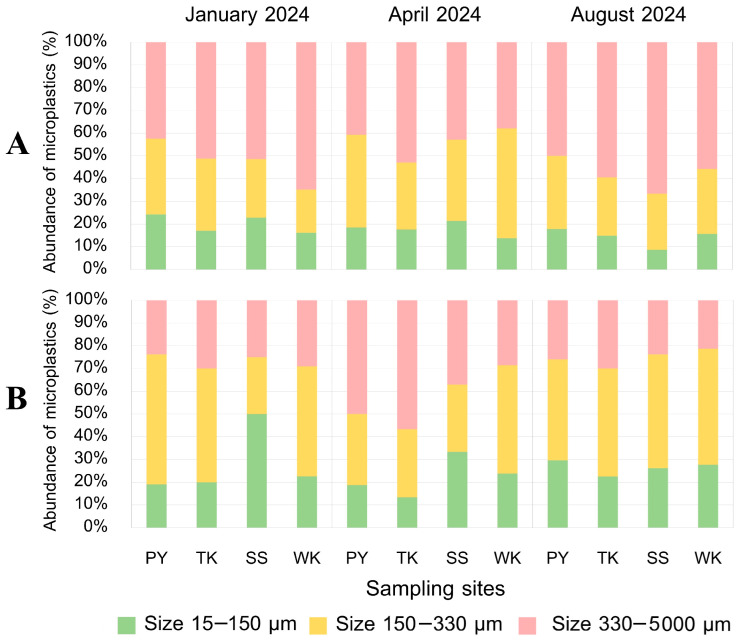
Composition of MP size range (%) (**A**) internally and (**B**) externally along Rayong province: Phayun—PY, Takuan—TK, Suanson—SS, Wangkaew—WK.

**Figure 8:**
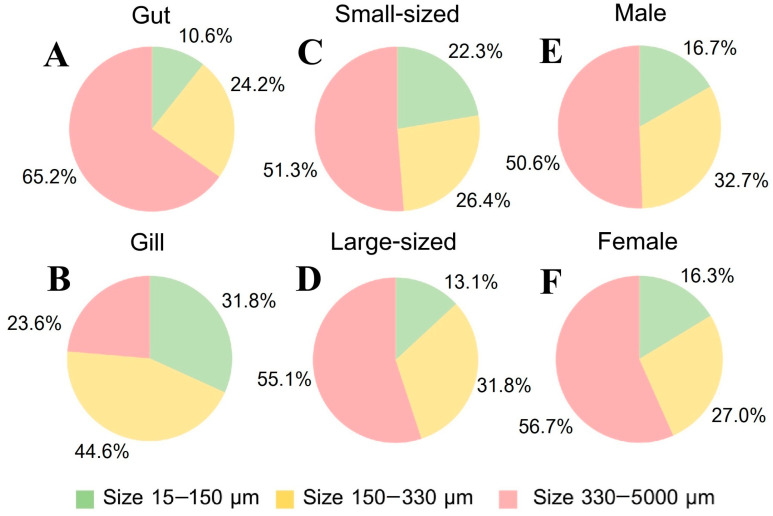
Size composition of MP internal contamination (%) in (**A**) gut and (**B**) gill of *P. pelagicus;* and (**C**) small-sized, (**D**) large-sized, (**E**) male, and (**F**) female *P. pelagicus* in Rayong province.

Our study highlights that MPs in *P. pelagicus* are predominantly larger in size, particularly those ranging between 330–5000 µm, which were consistently the most common across all seasons and sampling sites. This size category was notably more prevalent in internal contamination, especially in the gut, reflecting that larger MPs are more frequently ingested by crabs, as larger particles might be less likely to degrade or be filtered out of the digestive tract. Their prevalence in internal contamination indicates these MPs are effectively ingested and retained within the digestive systems of blue swimming crabs. While compared to external contamination, MPs sized 150–330 µm were more frequent, with the exception of SS, where smaller MPs (15–150 µm) were predominant (Figure 7). Seasonal variations in MP size distribution were observed, with larger MPs being more common in April, while MPs sized 150–330 µm were more prevalent in January and August. This variation might be influenced by seasonal changes in MP sources, such as an increased runoff during rainy seasons, and waste management, which could introduce different sizes of MPs into the environment. Site-specific trends showed that larger MPs were dominant in internal contamination across all sites, while external contamination varied, with different sites showing preferences for either smaller or larger MPs. A tissue analysis revealed that the gut primarily accumulated larger MPs (330–5000 µm), while the gills had a higher proportion of MPs sized 150–330 µm (Figure 8). This indicates that different tissues filter and retain MPs differently. The gut has a larger capacity for holding MPs, and its role in digestion could explain the accumulation of larger particles, while gills are involved in filtering water for respiration, and it may capture smaller MPs more effectively. This disparity seems to be linked to the anatomical position of the gill within the crabs and their pathway for gas exchange occurring by seawater flow through their narrow openings. This structure might prevent larger plastic particles from entering their gills [54]. According to a previous study, the size of MPs was found to be higher than *P. pelagicus* from 100–200 µm [55]. Additionally, the size of MPs in the internal body, especially in the range of 330–5000 µm (r = 0.506, *p* < 0.01) and 150–330 µm (r = 0.392, *p* < 0.01), was found to be related to size of the crabs, with larger crabs accumulating larger MPs and smaller crabs containing a lower composition of large-sized MPs. This could be due to larger crabs having a broader feeding range and being exposed to different environmental conditions compared to smaller crabs. It also underscores how MPs of different sizes may be selectively ingested and retained based on the size of the organism. However, the size composition of MPs between male and female crabs did not show significant differences.

#### 3.5.2. MP Shape

**Figure 9:**
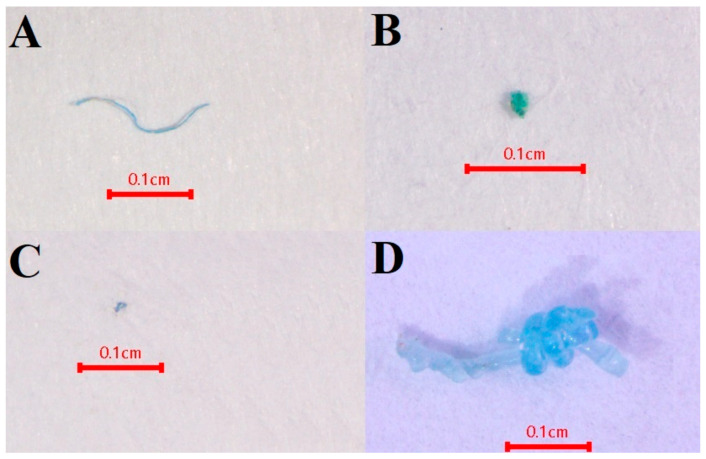
Shape of MPs: (**A**) fiber, (**B**) fragment, (**C**) film, and (**D**) net.

**Figure 10:**
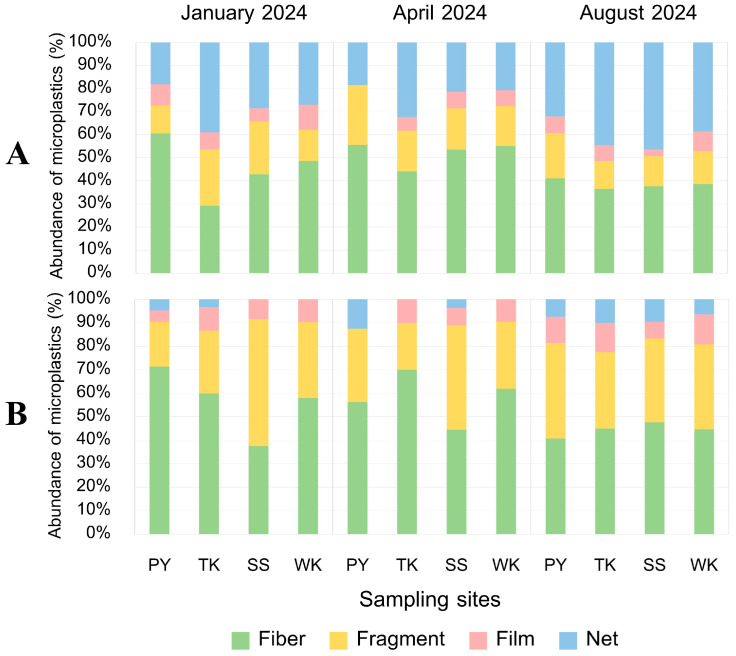
Shape composition of MPs (%) (**A**) internally and (**B**) externally along Rayong province: Phayun—PY, Takuan—TK, Suanson—SS, Wangkaew—WK.

Our study revealed that MPs in *P. pelagicus* were primarily found in four shapes: fibers, fragments, films, and nets (Figure 9). Fibers were the most prevalent type of MPs, observed in both internal and external contamination across all seasons, with a significantly higher abundance (Kruskal–Wallis, *p* < 0.05) compared to other shapes, accounting for 38.5% to 52.1% of internal MPs and 44.5% to 58.1% of external MPs (Figure 10). The net type was found in the second highest abundance, especially from the internal body, at 23.2% to 40.4% across all seasons, highlighting its dominance and indicating that fibers and nets were major components of MP pollution in *P. pelagicus*. The high contamination across sampling times and sampling sites indicates a widespread source of fiber contamination. Fibers are often associated with textiles, fishing gear, and industrial processes. The presence of fibers could be linked to common sources such as marine litter from fishing activities [56] and the breakdown of synthetic textiles from households [57]. Nets were predominantly found in the internal body, with a significantly higher abundance than the external body. In contrast, fragments were more frequently found externally, significantly higher compared to internal contamination. Films were found at a lower amount, showing similar abundance in both internal and external bodies. A sampling site analysis showed that fibers were the dominant shape at all sampling stations. PY had a high fiber prevalence in both internal and external contamination, while TK exhibited a higher proportion of nets internally and fragments externally. SS showed external contamination was predominantly fragments, while WK also had a high prevalence of fibers.

**Figure 11:**
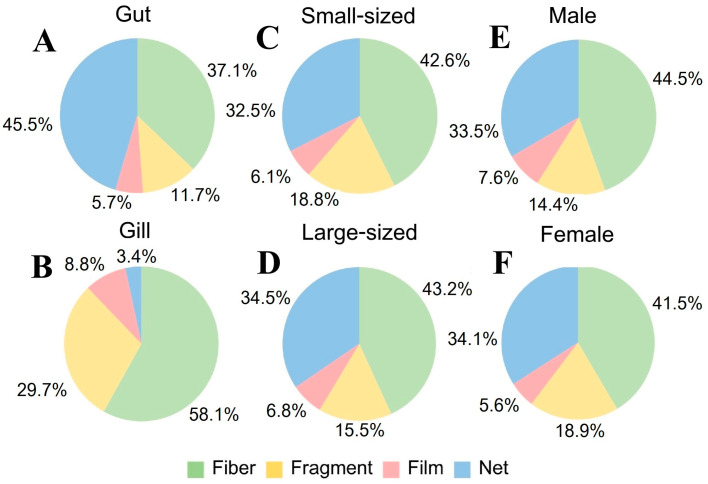
Shape composition of MPs from internal contamination (%) in (**A**) gut and (**B**) gill of *P. pelagicus,* and (**C**) small-sized, (**D**) large-sized, (**E**) male, and (**F**) female *P. pelagicus* in Rayong province.

The shape of MPs did not significantly vary with the size and sex of the crabs, indicating size and sex are not major factors influencing MP shape distribution. However, within tissues, fibers were the most dominant type in gills, while nets were the most dominant type in guts, with significant differences in their proportions compared to other shapes. There was a higher prevalence of nets and fibers in the gut (45.5% and 37.1%) and a high proportion of fiber and fragments in the gills (58.1% and 29.7%) (Figure 11). A variety of MP shapes show the disintegration of larger plastic debris into smaller particles within marine environments [58]. According to the results, the composition of MPs revealed that fibers were mostly prevalent, followed by net and films. This pattern of dominance by fibers was also observed in the environmental area along the coast of Rayong province [15,39]. Similarly, for *Carcinus aestuarii,* fiber was also found to be the dominant shape in the body [47]. On the other hand, fragments were the most common MP shape found in *P. armatus* [59]. Furthermore, for other marine fauna such as fishes from the Baltic Sea, we found MPs accumulated in the digestive tract and gills, with fiber being the most prevalent among the other forms [60]. This indicates that synthetic microfibers are widely contaminated in aquatic animals across various habitats and feeding strategies which can pose risks to the food chain, and to high-trophic-level organisms [61].

#### 3.5.3. MP Color

**Figure 12:**
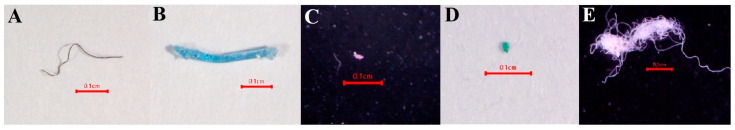
Color of MPs: (**A**) black, (**B**) blue, (**C**) red, (**D**) green, and (**E**) clear.

**Figure 13:**
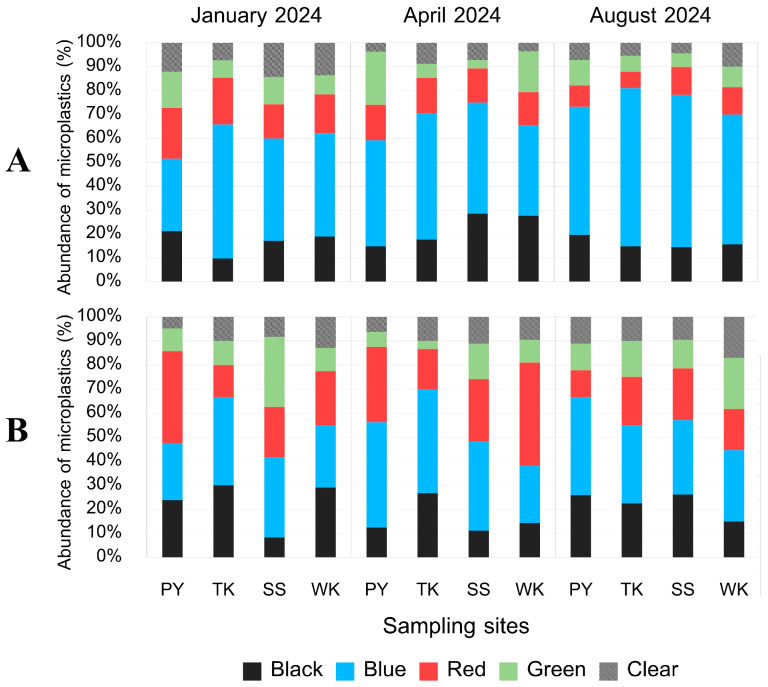
Color composition of MPs (%) (**A**) internally and (**B**) externally along Rayong province: Phayun—PY, Takuan—TK, Suanson—SS, Wangkaew—WK.

**Figure 14:**
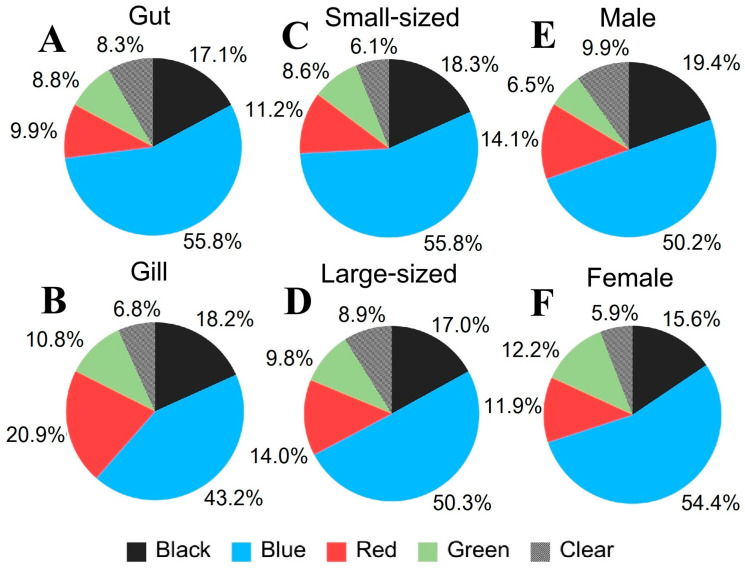
Color composition of MPs from internal contamination (%) in (**A**) gut and (**B**) gill of *P. pelagicus,* and (**C**) small-sized, (**D**) large-sized, (**E**) male, and (**F**) female *P. pelagicus* in Rayong province.

Our study indicates that four colors, black, blue, red, and green, along with clear MPs were detected in the MP samples (Figure 12), with blue MPs being the most prevalent. Blue MPs were consistently found in *P. pelagicus* across all seasons and sampling sites, both internally and externally (Kruskal–Wallis, *p* < 0.05), which related to the shape distribution, with fiber and net shape having significant internal contamination percentages in January, April, and August 2024. Black MPs were found exclusively in fiber shapes, while red and green MPs appeared in both fiber and fragment shapes. Clear MPs were mainly detected as fibers. Analyzing by sampling sites, blue MPs dominated the internal and external contamination at all sites. At Phayun station, blue MPs were the most abundant, followed by black, green, red, and clear internally, while red MPs were the second most common externally, followed by black, green, and clear. Takuan station showed a similar dominance of blue MPs, with black MPs being the second most common both internally and externally (Figure 13). Suanson station also had blue MPs as the most prevalent, followed by black and red internally, while red was the second most common externally. Wangkaew station exhibited blue MPs as the most common internally, followed by black, red, green, and clear, whereas red MPs were the most abundant externally, followed by blue, black, green, and clear. In terms of tissue accumulation, blue MPs were the dominant group in the gut of *P. pelagicus*, followed by black, red, green, and clear. Similarly, in gills, blue MPs were the most common, followed by red, black, green, and clear (Figure 14). Our study did not find a significant relationship between the color of MPs and the size or sex of *P. pelagicus*. The shape of MPs was also linked to their color; blue MPs were mostly found in fiber shapes, while black and clear MPs were predominantly fibers. Red and green MPs appeared only in fibers and fragments. According to a previous study, crabs rely on their vision to identify their food, which might be affected by the size and color of these particles. Due to the characteristics of MPs that seem to be similar in size to sand and resemble the color of their prey, this may cause MPs to appear as prey and sand to crabs. Crabs may mistakenly take these particles, resulting in mistaken consumption [43]. The dominant blue MPs indicate that they might be originating from common marine activities such as fishing, where blue nets and ropes are frequently used, and also from terrestrial waste. Recent studies have demonstrated that blue plastics are a general color of plastic extensively incorporated into synthetic textiles in our daily lives [62]. Moreover, broken fishing nets from fishing activities, which are usually made from blue fiber MPs, were mostly contaminated in the guts of organisms [56]. The presence of black, red, green, and clear MPs, though less prevalent, also points to various sources, including textile fibers, packaging materials, and other consumer products.

#### 3.5.4. MP Polymer

**Figure 15:**
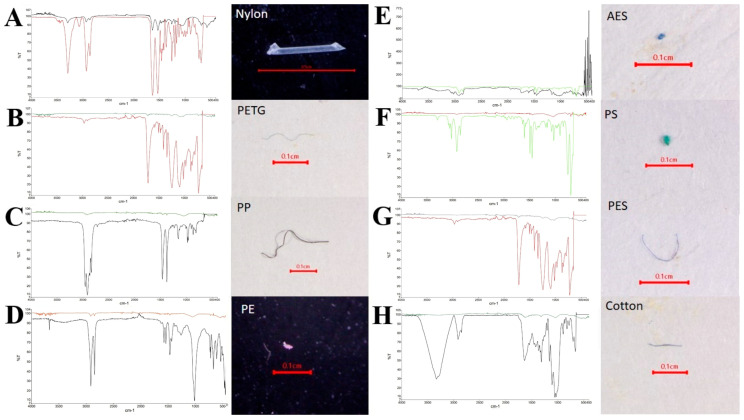
Polymer type of MPs in *P. pelagicus:* (**A**) nylon, (**B**) PETG, (**C**) PP, (**D**) PE, (**E**) AES, (**F**) PS, (**G**) PES, and (**H**) natural fiber—cotton.

**Figure 16:**
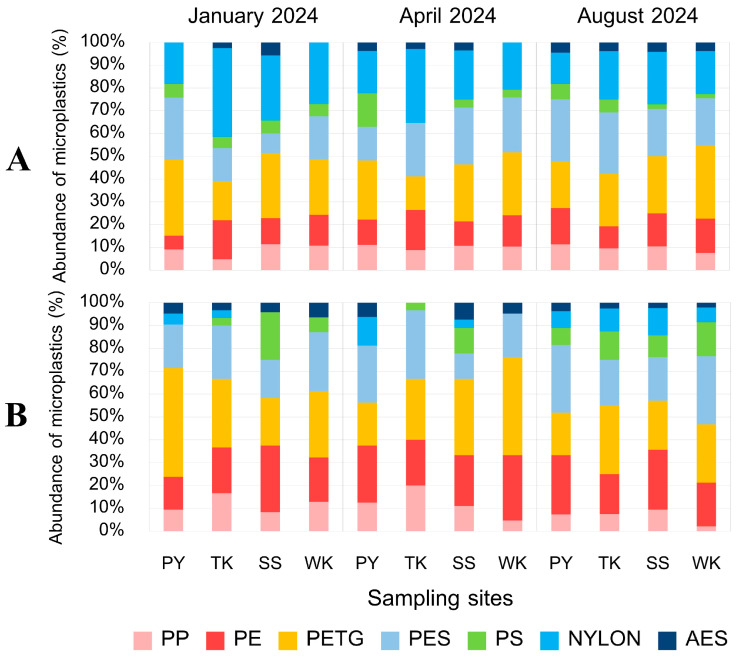
Polymer composition of MPs (%) (**A**) internally and (**B**) externally along Rayong province: Phayun—PY, Takuan—TK, Suanson—SS, Wangkaew—WK.

**Figure 17:**
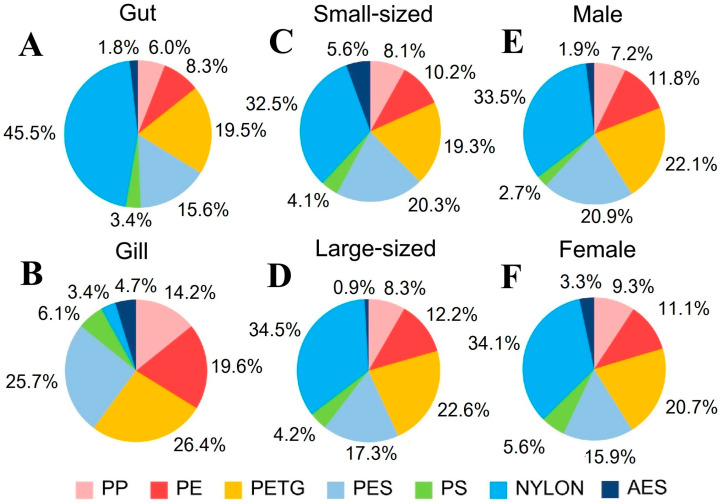
Polymer composition of MPs from internal contamination (%) in (**A**) gut and (**B**) gill of *P. pelagicus,* and (**C**) small-sized, (**D**) large-sized, (**E**) male, and (**F**) female *P. pelagicus* in Rayong province.

The FTIR verification results identified seven types of MP polymers (Figure 15), showing a varied distribution across shapes, sampling sites, and tissues. Nylon was mainly found in net shapes, while PETG and PES were primarily found in fiber shapes (Figure 16). PE and PP appeared as fragments and fibers, and PS and AES were found mainly in fragments. These polymers were detected in both internal and external contamination, with distinct patterns at different sampling sites. At the PY site, PETG was the dominant polymer both internally and externally, followed by PES. TK had the highest internal contamination of nylon, while PETG dominated externally. The SS had PETG as the main internal contaminant, while PE was most prevalent externally. WK had PETG as the primary polymer for both internal and external contamination, with different second most abundant polymers for each. Significant differences in the polymer composition were found between the gut and gill tissues of *P. pelagicus*. Nylon was the most common in the gut, while PETG was predominant in the gills (Figure 17). The shape distribution revealed that fibers were mainly PETG and PES, followed by PP and a small proportion of PE, in various colors. Polystyrene and AES were found only in fragment shapes, while films were only PES, and nylon was only in net shapes. Cotton fibers were also detected but were not considered MPs. Nylons are widely used in fishing gear such as webbing nets and ropes for their strength and resistance [63,64], which was predominantly observed in net shapes. This indicates that fishing activities, especially from crab gillnets, are a significant source of nylon contamination in blue swimming crabs. Durable materials like nylon were used as webbing nets for crab gillnets, including the float line and sinker line, with polyethylene and polypropylene rope [40]. They were designed to withstand harsh coastal environments; their degradation over time can contribute to MPs pollution. Although it is a durable material, these monofilament gillnets have a short lifespan, averaging around 1 to 2 months [65].

It means that they frequently need to be replaced. Broken gillnets and lines in contact with the harsh current during the southwest monsoon can release large amounts of MPs into the environment and these MPs can accumulate in organisms. The input of these MPs from damaged fishing gear will increase the load of MP debris, which exacerbates the contamination problem in the environment. PETG is a thermoplastic formed by polyethylene terephthalate (PET) and ethylene glycol with a high impact resistance and ductility, which are commonly used in packaging, synthetic textiles, and various industrial applications [66], and also in 3D printing material [67], while polyester was mainly identified as fibers, pointing to sources like deteriorated textiles from household waste [68]. Polypropylene is often found in packaging and household products [69], while polyethylene is widely used in packaging items like shopping bags, plastic bottles, and containers with highly utilized materials in the industry [70,71]. These PE and PP polymers are also commonly used in the production of fishing nets [72], which are observed as both fragments and fibers. Meanwhile, polystyrene is used in domestic plastic products, which are usually used in disposable packaging [73,74], and poly (Acrylonitrile Ethylene Styrene) is used in automotive parts. These PS and AES polymers were primarily detected in the fragment shapes. Their presence indicates origins such as discarded packaging from terrestrial waste. Our study was limited by a lack of environmental samples, such as water and sediment from fishing ground areas, which could provide further insights and potentially clarify the MP situation in Rayong province. However, examining MPs from external biota could define these organisms as confronting the contamination of MPs in their environment. Our study was also limited by the small sample size at each sampling site (N = 12), with subgroup comparisons having only three individuals (N = 3). This low sample size may have reduced the statistical power. Future studies should aim for larger sample sizes to increase the statistical power and validate our findings. Although a hot needle test was performed on all visually identified particles to differentiate them from natural materials, the discriminative power of this method is still limited, and it only separated the plastic and non-plastic categories. Although identifying MP polymer types by FTIR was performed on 10% of total MPs in crab samples, a hot needle test might also assist in verifying MP types within the study. The subsampling uncertainty of all polymer levels was also described. A total of 99 MP particles (approximately 10%) were randomly selected and analyzed using FTIR to determine the polymer composition. To propagate the subsampling uncertainty into the overall dataset, nonparametric bootstrap resampling (5000 iterations) was applied to subsamples. The result indicated 95% confidence intervals (CIs) for polymer proportions were scaled to the total particle count observed microscopically. The extrapolation estimates are indicated in Table S1. Importantly, the reported counts for all polymers fell within the 95% confidence interval derived from the subsample analysis, confirming that the extrapolated estimates are consistent with the uncertainty associated with subsampling.

## 4. Conclusions

Microplastic contamination in blue swimming crabs (*Portunus pelagicus*) was consistently detected across all sampling stations, indicating that microplastics are widespread in the fishing grounds. Contamination was higher in internal organs than external surfaces, with the gut being the most affected tissue. Seasonal variation was evident, with the highest contamination observed in August 2024. Most of the detected microplastics were fibers, predominantly blue, with polyethylene terephthalate glycol, nylon, and polypropylene being the major polymers. Due to the separation procedure, which only used an NaCl solution, higher-density polymers will settle down to the bottom of the solution and could not be recovered. However, this procedure was adjusted by collecting the settled solution separately and filtering it, which could recover higher-density polymers like polyethylene terephthalate glycol and nylon in samples. These findings highlight that anthropogenic activities, including household wastewater rich in synthetic fibers and the use of nylon crab gillnets, are key contributors to microplastic contamination in this area. To mitigate this issue, efforts should focus on improving waste management practices, reducing fiber release from laundry, and developing more durable and eco-friendly fishing gear. Future research should investigate the specific sources of microplastics at each site and assess their long-term impacts on marine organisms and ecosystems. Policy interventions and public awareness are also crucial to reducing plastic pollution and safeguarding marine biodiversity and food safety.

## Figures and Tables

**Table 1:** Summary of the characteristics of blue swimming crab samples.

SITE	PY	TK	SS	WK
Crab samples (individual)	36	36	36	36
Carapace width (cm)	11.24 ± 0.97	11.02 ± 1.08	11.33 ± 0.98	11.15 ± 0.92
Carapace length (cm)	5.52 ± 0.54	5.38 ± 0.61	5.61 ± 0.60	5.53 ± 0.53
Total weight (g)	103.11 ± 25.28	97.71 ± 33.77	107.15 ± 27.15	98.89 ± 26.18
Gut weight (g)	1.01 ± 0.53	1.02 ± 0.51	1.01 ± 0.48	1.17 ± 0.56
Gill weight (g)	4.33 ± 1.19	4.15 ± 1.66	4.71 ± 1.30	4.54 ± 1.34
Total external detection (individuals)	22	23	23	22
Total internal detection (individuals)	25	29	24	26
Total external MPs (items)	64	100	93	99
Total internal MPs (items)	116	149	132	136
MPs in gut (items)	82	107	100	96
MPs in gill (items)	34	42	32	40

Note: Phayun—PY, Takuan—TK, Suanson—SS, Wangkaew—WK.

## Data Availability

The data will be made available upon request.

## Supplementary Materials

The following supporting information can be downloaded at: https://www.mdpi.com/article/10.3390/toxics13100813/s1, Figure S1: Interview questionnaire form; Table S1: Summary of estimated polymer counts and proportions with 95% confidence intervals (CIs) propagated from subsampling uncertainty.

## Abbreviations

The following abbreviations are used in this manuscript:

MPs	Microplastics
PY	The station is called “Payoon”
TK	The station is called “Takuan”
SS	The station is called “Suanson”
WK	The station is called “Wangkaew”
PP	Polypropylene
PE	Polyethylene
PETG	Polyethylene Terephthalate Glycol
PES	Polyester
PS	Polystyrene
AES	poly (Acrylonitrile Ethylene Styrene)
