# Supplementary material for: The Contamination of Microplastic Debris in Blue Swimming Crab Portunus pelagicus (Linnaeus, 1758) from Artisanal Fisheries in the Eastern Gulf of Thailand
Source: Toxics. 2025 Sep 24;13(10):813. doi: 10.3390/toxics13100813 (PMC12568171; doi:10.3390/toxics13100813)
Supplement: Supplementary file 1 [file toxics-13-00813-s001.zip › toxics-3876160-supplementary.pdf]

Table S1. Summary of estimated polymer counts and proportions with 95% confidence intervals (CIs) propagated from subsampling uncertainty.

Polymer	Reported count	Proportion %	95% CI (%)
PETG	214	24.1	16.2–32.3
Nylon	199	22.4	14.1–30.3
PES	179	20.1	12.1–28.3
PE	139	15.6	8.1–22.2
PP	79	8.9	4.0–15.2
PS	52	5.8	2.0–11.1
AES	27	3.0	0.0–7.1

Figure S1. Interview questionnaire form

Interview questionnaire form (Gillnets)

****

Sample NO. ……………………………

Date……………………………………….

1. Name of fisherman ……………………………… Name of sampling site ……………………………
   Address……………………………………………………Phone (optional)…………………………………….
2. Vessel data
   2.1 Vessel Name……………………… License No. ………………… Length of vessel……………….
   2.2 Engine ☐ No engine ☐ Longtail engine ☐ Inboard engine
   2.3 Brand……………………………………………. Power (Hp)……………………………………………….
   2.4 Type of Fuel ☐ Gasoline ☐ Diesel ☐ Others…………………………………………………….
   2.5 Equipment on board
   ☐ GPS ☐ Sounder ☐ Radio ☐ Winch ☐ Mobile phone ☐ Others…………………….
3. Target species
   ☐ Crab ☐ Shrimp ☐ Fish ☐ Others………………………
4. Fishing operation
   Methods of net deployment ☐ Parallel ☐ Perpendicular ☐ Others……………………
   Number of lines……… Distance between line (m)…….. Nets per line (pack) ………..
   Deployment duration………………☐ Minutes ☐ Hours ☐ Days
5. Gillnets characteristics
   Twine type ☐ Nylon ☐ PE ☐ Others……………Twine Size (D/Tex)…………………………
   Mesh size……….☐ cm. ☐ inch. Net depth……… Net Length per sheet (m)…………..
   Life span…………☐ Month ☐ Year Number of lost nets……………….☐ Sheet ☐ Pack
6. Fishing effort
   No. of nets employed (pack)…………………………Net per pack (sheet)……………………..
   Total Stretched Length (m)……………………………………………………………………………….
   Net retrievals per trip (time)………………………Duration of trips (days)…………………….
7. Fishing ground
   Travel time to the fishing grounds………………………………………… ☐ Minutes ☐ Hours
   Distance from shore……… ☐ Km. ☐ Nautical mile Depth of water…….. meter
   Fishing departure time…………………………… Fishing arrival time…………………………

Fishing ground coordinates	
Latitude	Longitude

8. Environment around fishing ground
   ☐ Fishing communities ☐ Restaurant ☐ Industry ☐ Tourism ☐ Others…………………..
9. Remark………
10. Species composition and total catch weight…………………………………………………………Kg

Species	Size (cm)	Weight (kg)	Price (baht/kg)
